# How did UK social distancing restrictions affect the lives of women experiencing intimate partner violence during the COVID-19 pandemic? A qualitative exploration of survivor views

**DOI:** 10.1186/s12889-023-14987-3

**Published:** 2023-01-18

**Authors:** A. R. McKinlay, Y. R. Simon, T. May, D. Fancourt, A. Burton

**Affiliations:** Research Department of Behavioural Science and Health, Institute of Epidemiology & Health Care, University College London, 1-19 Torrington Place, London, WC1E 7HB UK

**Keywords:** Intimate partner violence, Domestic violence, Public health, Mental health, Wellbeing, Social support

## Abstract

**Background:**

Increased numbers of domestic abuse cases were reported at the start of the COVID-19 pandemic. Many people experiencing abuse faced barriers to seeking support with service closures affecting the sector. Available evidence suggests women are overrepresented in the reported cases of intimate partner violence (IPV) and we aimed to learn more about how their lives were impacted by social distancing restrictions.

**Methods:**

We conducted an online qualitative interview study, using reflexive thematic analysis. Interviews were conducted between April 2021 and March 2022. 18 women in the UK with past experiences of IPV provided informed consent and participated in this study.

**Results:**

During the analysis, we identified five themes relating to the impact of lockdown restrictions on participants’ lives, including: (1) Lockdown meant being confined to a place where abuse was escalating, (2) Barriers to accessing support, including “cancelled” services and missed opportunities to intervene during interactions in lockdown with frontline workers. (3) Increased feelings of fear, isolation, and loss of control, particularly during the early stages of the pandemic from the combination of abuse and pandemic-related changes to daily life. (4) Some forms of support were more accessible during the pandemic, such as provision of online psychological support and social groups. Participants also accessed new forms of support for the first time during the pandemic, in some cases sparked by posts and content on social media about abuse awareness. (5) For some, psychosocial wellbeing transformed during the pandemic, with several participants using the word “freedom” when reflecting on their experience of simultaneously escaping abuse and living through the COVID-19 pandemic.

**Conclusions:**

In this study, we explored the views of female survivors of IPV in the UK during the COVID-19 pandemic. Our results highlight the importance of combined public awareness campaigns and community intervention points for victims to safely seek help during social distancing restrictions. Having the time and space to reflect on healing after escaping abuse was described by women in our study as a benefit from their lives in lockdown, which is a factor that could be incorporated into future initiatives developed to support people subjected to violence and abuse.

**Supplementary Information:**

The online version contains supplementary material available at 10.1186/s12889-023-14987-3.

## Introduction

After first being detected in 2019, coronavirus disease (COVID-19) caused by SARS-CoV-2 spread rapidly across the world until it was declared a global pandemic by the World Health Organisation in March 2020 [1]. The pandemic necessitated governments to implement social distancing restrictions in order to limit the spread of the virus. Throughout England, this meant that citizens in non-keyworker roles were ordered to “stay at home” under “lockdown” (see Supplementary file 1). People with pre-existing health conditions were identified as extremely clinically vulnerable with additional “shielding” guidance to protect themselves against serious infection from the virus. These restrictions resulted in safety concerns because the incidence of domestic violence and abuse was feared to rise [2]. Not everyone feels safe at home, and so, the stay at home guidance created a secondary problem for people who were living with violence and abuse when the pandemic was declared.

Intimate partner violence (IPV) is an indiscriminate crime; it affects people of all genders, all ages and all backgrounds, in all corners of society [3, 4]. The socioeconomic cost of domestic abuse (DA) in the UK alone is estimated at £66 billion [5]; but in reality, the true cost of abuse can never be quantified. Although all people are at risk of experiencing violence and abuse, women are overrepresented in reported cases of IPV [6] and their experiences of this crime warrant specific focus [7]. On average in Britain, women are more likely than men to earn less [8] and have more unpaid care responsibilities [9]. Research suggests that women in the UK experienced less equality during COVID compared with pre-pandemic times [10, 11] owing to these pre-existing gender inequalities. Women are more likely to experience fatal consequences of IPV such as femicide, and are more often victims in reports of physical abuse, sexual violence, and coercive control cases [6, 12]. IPV has far-reaching and long-lasting consequences; survivors of IPV are twice as likely to develop depression and anxiety compared with the general population [13].

Reports of violence and abuse often increase during times of adversity, such as natural disasters [14], emergencies [15], and epidemics [16]. Various explanatory factors have been proposed including economic collapse, closure of violence prevention initiatives and social support services, breakdown of social networks and heightened psychological distress [17–19]. The frequency of IPV [20, 21] reports rapidly increased around the world after the first COVID-19 lockdown restrictions commenced. Reports to crisis services have suggested more severe cases of abuse [22, 23], particularly physical IPV [21]. The first week of lockdown in the United Kingdom (UK) saw a 25% increase in calls to abuse hotlines and a 150% increase in DA website visits [24], which might have been indicative of increasing tensions in homes and pressurised local services [25]. However, some research suggests a decrease in reporting of IPV [26], due to difficulties in reporting crimes and seeking help under lockdown, due to digital poverty, financial hardship, lack of childcare, service closures, COVID fears, or being under the watch of an abuser [22, 27]. Women’s support services pre-lockdown have been crucial in reducing victimisation and improving the overall well-being of survivors [28]. However, under lockdown restrictions, many UK services for IPV were closed, discontinued and in some cases, moved online [29].

Emerging research into violence against women during COVID-19 has highlighted concerning findings regarding the reality of life for people experiencing IPV in lockdown [30], suggesting this group require specific focus in efforts to support the health of the public. A number of qualitative studies conducted internationally in the early stages of the pandemic reported a deterioration in mental health amongst IPV survivors, including worsening of existing mental health concerns (such as depression and anxiety) and heightened financial distress [31, 32]. Little is known about the mental health impacts or support needs reported by women in the UK experiencing IPV in the various stages of the COVID-19 outbreak. Women’s abuse support services have been identified as vital in providing shelter and counselling, although in some instances, victims report that shelters under isolation can cause re-traumatisation [32], which means alternative means of ensuring safety are essential in cases of future pandemics. Victims and survivors have also mentioned difficulty in receiving remote women’s health services during the COVID-19 pandemic due to lack of accessibility or feeling uncomfortable [23, 33], and therefore, alternative means of remote psychosocial support warrant investigation.

At the time of writing, no published qualitative studies to our knowledge had been undertaken with a focus on female survivors of IPV in the UK, which saw one of the worlds’ longest lockdowns during the pandemic [34]. There remains a need for further qualitative research into the impact of lockdown measures in order to explore improvements to service provision and public policy. We asked the following research questions to guide the conduct of the research: (1) How did social distancing restrictions affect the lives of women experiencing IPV during the COVID-19 pandemic? (2) What were the social and mental health impacts of pandemic-related changes on women experiencing IPV? (3) How were women’s access to support for IPV affected by pandemic-related disruptions?

## Methods

The research was conducted as part of the University College London (UCL) COVID-19 Social Study (CSS), which was a large mixed methods study, exploring the social and mental health impacts of the pandemic [35]. Ethical approval was obtained from the UCL Research Ethics Committee (14,895/005).

### Recruitment and sample

Interviews took place between April 2021 and March 2022, with the majority of interviews occurring between October 2021 until early 2022. There were two sampling strategies employed during recruitment; we first employed convenience sampling methods, whereby, we advertised the study through specialist third-sector services providing domestic abuse support. We invited women who were “safe from abuse” (i.e., not currently experiencing abuse or living with someone carrying out abuse) to contact us if they were interested in participating. This was to ensure that women contacting the researchers were less likely to be put at-risk by contacting us. We were cognizant that during the pandemic many abuse victims were not able to discuss confidential matters safely or talk freely without risk of being overheard whilst still living with an abusive person [36]. After all social distancing restrictions had ended in July 2021, we began to display notices about the study on more widespread public platforms such as the CSS social media page and newsletter (reaching ≥ 3000 people). During this time, we employed more purposive sampling methods improve diversity within the group of participants in terms of age and ethnicity.

Our study eligibility criteria were female, not currently experiencing abuse but had experienced any form of abuse from a partner during the pandemic, aged 18 or over, and no longer living with an abusive partner. Those who were interested and identified themselves as eligible contacted the lead researcher (AM) for more information. The lead researcher sent potential participants an Information Sheet, a study Screening Form (see Supplementary file 2), and an offer to answer any questions. After screening, 18 women were eligible and agreed to participate, while 7 women were not eligible to participate (5 were not eligible based on living circumstances and 2 were ineligible based on experiences of abuse that occurred before rather than during the pandemic). Once study eligibility had been confirmed and participants had read the information sheet, a consent form was sent to participants, which was signed by the lead researcher and participant. After completing a written informed consent form, participants completed a demographics questionnaire, then an interview time and day was arranged.

### Interview procedure

Participants attended a one-to-one remote video or telephone interview, conducted by one of two experienced female qualitative researchers (AM, AB) with expertise in mental health research. Interviews followed a semi-structured format, with several specific question prompts (see Table 1 for examples). The interview process began with the lead researcher introducing themselves and the COVID-19 Social Study more broadly, before explaining the aims of the current study and focus on support for women experiencing IPV during the pandemic. The researcher then explained the topics that were of interest: life before the pandemic, impact of social distancing restrictions, support for abuse, social life, mental health, thoughts about the future. The researcher verbally noted key points in the written consent form including the limits of confidentiality (i.e., if immediate risk of harm was disclosed during an interview).

**Table 1:** Topic guide examples

Questions
1. Could you tell me about any forms of support you accessed to help you with domestic abuse?
2. Was there any impact of the pandemic on your ability to receive help?
3. Is there anything that support services could have done better?

The interview procedure was conducted carefully and variably, with priority placed on the pace and content set by participants rather than following a uniform procedure. Authors AM and AB met weekly throughout the study to debrief, reflect, and discuss preliminary impressions of the interviews so that any questions or approaches could be tailored and refined as the study progressed. In some cases, only one or two topics were discussed during an interview with minimal questions or prompts asked by the interviewer, in favour of allowing the participant space to tell their story. For interviews that were more semi-structured, questions asked by the interviewer were open-ended, with prompts used such as “How do you feel about the changes that have been brought about by COVID-19?” (See Supplementary File 3). Participants were not asked about specific experiences of abuse, nor were these topics explored in detail if mentioned by participants during the conversation. In some cases, interview topics and question examples were sent to participants beforehand if this was requested so that participants could review topics ahead of their interview. Participants were offered a £10 voucher as compensation for their time and participation in the research.

Interview duration was on average one hour and 10 min, ranging from 16 min to two hours. Interviews were audio recorded and then transcription conducted by an external company with whom the research team have a data sharing agreement. All identifiable details (e.g., names, locations, etc.) were redacted. Audio files and transcripts were saved separately from any other information within the UCL Data Safe Haven, a certified secure data storage platform.

### Qualitative data analysis

We conducted a reflexive thematic analysis informed by Braun and Clarke [37], to extract rich and detailed data from interview transcripts. Thematic analysis was chosen to analyse the interview transcripts, as it provides a ‘starting point’ for analysing complex research data [38], whilst allowing the researcher flexibility with concept synthesis [39]. We did not seek “thematic saturation” in the study analysis, as this concept contains the assumptions of realism [40], which were not the principles drawn upon in this study. Instead, our research methodology was informed by principles of feminist epistemology [41] and critical realist ontology [42], whereby, we sought to amplify and validate the voices of those who have faced inequality and oppression from abuse.

After fieldwork and interview transcription were completed, files were imported and managed using NVivo version 12 [43]. AM read and coded all interview transcripts; however, to enhance depth of understanding of the data and subsequent themes amongst the study team, AB and YS also read a subsample of transcripts. AM coded passages of text that were pertinent to the study research questions. Some additional relevant contextual information (i.e., whether someone was in temporary housing, had informal caring responsibilities, or was struggling with substance misuse) were also coded. AM labelled all initial codes using language and terms that were aligned as closely as possible to the participants’ words. After all transcripts had been coded, AM organised the codes into groups, based on what she felt were similar topics, for instance, mentions of abuse becoming “more frequent” or “more intense” were grouped together and later labelled: “abuse escalated during the pandemic.” These groups of topics were eventually used to inform theme development, which were labelled based on AM’s interpretations. For instance, the code “abuse escalated during the pandemic” helped to form theme one, called: “Stay at home orders meant being confined to a place where abuse was escalating.”

After the first round of coding was complete, AM presented preliminary themes to study co-authors (AB, YS, TM) and peers working on the CSS, to help “sense-check” the topics identified [44]. Presenting to peers in this way can help to become further immersed in the data and improve the depth of insights garnered from the dataset [40]. This step was undertaken to elicit co-author feedback on initial theme labels, to check that these were coherent and relevant to study aims. AM then selected themes that responded most directly to the research questions, as opposed to selecting analysis themes that were frequently endorsed by participants [40]. AM presented the themes a second time to study co-authors (AB, TM), and then finalised the list of themes and subthemes.

In the Results section, we present quotes that are intended as a representation of subthemes, with full quotes presented in Supplementary File 4. Some quotes have been shortened in-text for brevity and clarity.

## Results

### Characteristics of participants

Participants’ ages ranged from 24–61 (41 years on average), with most participants identifying as White British (see Table 2). Participants had mostly completed university-level education. Ten women were living with their partner at the time that abuse was occurring. Nine women had caring responsibilities at the time of interview. Eight women lived with their young children during and prior to the pandemic, or their grown children had moved back into the family home because of the pandemic (for instance due to university closures). Six participants had a physical health condition (including diabetes, asthma, and high blood pressure) and eight had a mental health condition (including depression, anxiety, post-traumatic stress disorder (PTSD), and eating disorder).

**Table 2:** Participant demographic characteristics

***Demographic category***		***Result*** *n* = *18*
**Age**	20–29	5
	30–39	4
	40–49	3
	50–59	5
	60–69	1
**Ethnicity**	White British	14
	White Other	2
	Indian	1
	Pakistani	1
**Number of children**	0	10
	1	5
	2	2
	3	1
**Education**	Postgraduate	10
	Undergraduate	6
	A-Levels or equivalent	1
	Post-16 vocational	1
**Employment**	Fulltime	6
	Part-time employed	5
	Self-employed	3
	Unemployed / unable to work / seeking work	3
	Retired	1
**Relationship status**	Single	11
	Divorced / separated	6
	Long distance relationship	1
**Living situation**	Lives alone	9
	Lives with children, housemates and/or family	6
	Temporary housing	3

### Qualitative analysis

**Fig. 1:**
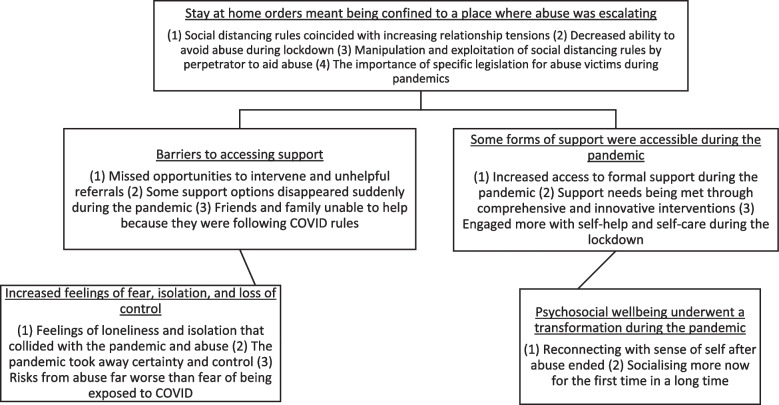
Summary of qualitative themes and subthemes generated during the analysis

We generated 5 themes relating to the effect of social distancing restrictions on women’s lives, including: (1) Lockdown meant being confined to a place where abuse was escalating, (2) Barriers to accessing support. (3) Increased feelings of fear, isolation, and loss of control. (4) Some forms of support were more accessible during the pandemic, (5) Psychosocial wellbeing transformed during the pandemic (see Fig. 1).

#### “Stay-at-home” orders meant being confined to a place where abuse was escalating

Many women in the study witnessed an increase in the frequency and intensity of abuse being carried out in their homes with the announcement of lockdown restrictions. They described increasingly stressful living situations, where usual outlets of frustration for all parties within the household had been removed and resources for adopting healthy coping strategies (such as going for a walk or seeing a friend) were restricted.

##### Social distancing rules coincided with increasing relationship tensions

Many participants who were living with an abusive partner at the time of the pandemic declaration described how the introduction of social distancing restrictions combined with other factors contributed to pent-up stress within their homes.

*“There was too much stress, and also the pandemic and then we also got ill with coronavirus. So, there was lots and lots of stress at the same time and accumulating financial stress. All that came out into how he interacted with me, how he interacted with the kids, becoming a bit more controlling, becoming a bit more particular about how things are done, with more arguments, more pressure and more control.” ID9, aged 20–29, separated, parent, living in temporary accommodation.*

For some, the impact of the pandemic on daily life pre-empted adoption of unhealthy coping behaviours within the household, resulting in arguments that were more likely to escalate, putting further strain on relationships.

*“Very quickly, my husband was drinking very heavily, staying up very, very late at night, and he was clearly, really struggling with the loss of his work, his identity, his income… He just found the whole pressure, he couldn’t communicate, at all, and what it did was highlight all the cracks that were there before the pandemic. Every little thing was suddenly magnified by the close proximity, by the restrictions, by the changes and everything else. Just everything fell apart, very, very quickly.” ID7, aged 50–59, divorced, parent, living alone.*

##### Decreased ability to avoid abuse during lockdown

One of the main topics that participants discussed in relation to their lives in lockdown was that the usual “escape routes” they had built into their lives prior to the pandemic to avoid an abusive partner were no longer accessible under social distancing restrictions.

*“I was less able to build things into my day, because he was here, in my face all the time, and if he needed to talk, then we talked and other things would take a back seat.” ID4, aged 50–59, separated, no children, living alone.*

Being able to go to work, go for a walk or to the gym, spend time with friends, or physically attend a support service for IPV, were routes that participants had previously utilised to avoid abuse at home. However, this all had to stop because of the pandemic, which meant more time spent at home and potentially at risk. Not having the ability to escape or access the usual routes to avoid abuse or receive support meant some participants felt exposed to risk of further abuse.

*“Those cycles [of abuse] were happening so quickly, during the pandemic, it just really, really built up, and because I had no way of escaping it, no way of going to work and just relaxing, I was tense the whole time.” ID10, aged 20–29, single, no children, living with family.*

A group of participants described feeling trapped in their homes prior to the pandemic because of abuse. These feelings of being metaphorically trapped at home escalated during the pandemic, due to the length of social distancing restrictions, as multiple national lockdowns came into effect.

*“As the lockdown extended, I became very trapped. I was trapped by the crime, and I’m still trapped… The pandemic doubled it really. Mainly because there was no escape.” ID2, aged 60–69, single, no children, living alone.*

Although UK rules permitted victims of abuse to leave home for reasons relating to abuse, delays in sales of homes and separation proceedings meant that some participants had to stay for longer in their accommodation with their abuser than they would have otherwise chosen.

*“We had a buyer, we put it on the market in November, and we had a buyer, very quickly, and it was meant to go in January. So, I thought I’ll stay here until it goes, and then that fell through, and then the abuse started again.” ID10, aged 20–29, single, no children, living with family.*

There were also “invisible barriers” and family-related pressures that participants described as a reason for not being able to leave an abusive household during the pandemic. For instance, some had family move in with them during the lockdown and they did not want to cause a family rift or upset the children by leaving:

*“And I told…my soon to be ex-husband, that this was going to end. We were going to end. But we needed to see the boys through… Just logistically, no matter how bad it was, I just couldn’t face shuffling around.” ID3, aged 50–59, separated, parent, living with children.*

##### Manipulation and exploitation of social distancing rules by perpetrator to aid abuse

Several participants described the ways in which perpetrators exploited social distancing legislation to carry out further abuse. For instance, wearing a mask to obscure identity whilst stalking, or controlling participants’ movements within the home.

*“[Social distancing rules were] all taken really literally, and it got to a thing where my partner was okay, ‘you can’t actually leave the house without me’… they were taken very seriously by my partner and kind of used as another area of control.” ID11, aged 30–39, single, no children, living with family.*

Rules around shopping at supermarkets and leaving the house alone were another reason some perpetrators rationalised their use of control*.* For several participants who were classified as clinically extremely vulnerable, they described uncomfortable and at times, hostile situations, where an abusive ex-partner seemed to deliberately disobey the guidelines to put them at risk. In other cases, conflicting interpretations and confusions around social distancing rules were the reason some arguments developed:

*“The only time it’s been an issue has been if he’s been thinking I haven’t been, in his view, been careful enough… if I walk along the street, I can’t wear a mask, he think[s] I should have worn a mask in the street as well. But I tried to explain I couldn’t see, because of the glasses, but he didn’t really, fully, take that on board, and that was a bit of a bone of contention.” ID4, aged 50–59, separated, no children, living alone.*

##### The importance of specific government guidelines for abuse victims and survivors during the pandemic

A key announcement in the UK during the first lockdown was the ability for victims to leave home in cases of DA: *“The pandemic has absolutely not helped with domestic abuse, until the government did allow domestic abuse victims to leave their home.”* ID2. However, the announcement that people could leave their homes in instances of abuse was not enough to protect all people, particularly because there were not always places to them to go if they did leave. Some participants called for plans for victims to have been announced more clearly from the outset of the pandemic to provide much needed reassurance and safety for people being subjected to abuse.

*“It was good hearing things on the news about you don’t have to follow these rules if you’re going through this, it came in quite late, perhaps too late… I kept thinking it’s all very well people saying you have to stay in your home, and stay with people you live with. I’m living with somebody who’s so vile to me, why am I not allowed to see anybody else, safely… that was very, very hard.” ID5, aged 40–49, divorced, parent, living with children.*

Events in the news such as #PartyGate where politicians were alleged to have attended illegal gatherings during lockdown were especially difficult to hear when participants reflected on what they had been living through at the time of the events. The announcement of the “support bubble” system was perceived as helpful because it meant that could see others for emotional and practical support. In some cases, this resulted in stronger connections with friends and family.

*“I see a lot more of my family because I’ve moved area. I think, in the second lockdown, I was able to bubble with my parents because I’m a single parent, so I saw a lot more of them then as well, which was nice.” ID12, aged 30–39, single, parent, living with children.*

#### Pre-existing and pandemic-related barriers prevented access to support

For those who had been living with abuse for many years prior to the pandemic onset, they described pre-existing barriers to support that were exacerbated by the pandemic, such as services that were not tailored to their needs or that they were not eligible for. While some participants received formal support from the NHS, government and third-sector organisations, most tried to access informal support via their friends and family networks. During the various lockdowns and lifting of restrictions, however, this proved to be a considerable challenge, leaving many feeling alone and turning to self-help strategies to support their mental health and wellbeing.

##### Missed opportunities to intervene and unhelpful referrals

Pre-existing gaps in services were exacerbated by the pandemic. In some instances, the help that women did receive was unsuited to their needs, based on demographic characteristics such as age or sexuality.

*“A lot of [services are] so directed to younger people, I wouldn’t feel comfortable. I looked into what’s available in the past, and just thought, I can support myself better.” ID8, aged 50–59, no children, living alone.*

Several women were discharged from the support they were accessing for being “too traumatised” or not “critical” enough: *“They sent me somewhere else, and they sent me to a couple of other places, one I’m not in critical need enough for, and the other I’m in too critical need for.”* *ID20*, *aged 30–39,* *parent, living with children*.

A group of participants said that they felt there were missed opportunities to intervene to divert the course of their experience of abuse during the pandemic. As one participant explained, she had interacted with the police on two occasions during the lockdown. On the first encounter the police officers did not take any action, but at the second police visit she was signposted to several forms of support and given the contact details of the officer, which helped her feel supported. Some participants felt that their local GP surgery could have provided an intervention point to access help for ongoing abuse during the pandemic.

*“The last time I saw a doctor there, she was really nice… she said, ‘if you ever just want to come in and have a chat, just come in and have a chat.’ And if that had been possible [during lockdown], that would have been my nearest version of having a family member, it was just that one-to-one, it wasn’t therapy, it would have just been nice to go in and say, oh, I’m having a hard time with this. And I would have done that, and there wasn’t that option, and I could have maybe dealt with it a bit sooner.” ID8, aged 50–59, no children, living alone.*

Some participants attributed online service provision as one of the reasons that many professionals seemed to miss the opportunity to help:

*“With the GP, they don’t like you to go to the doctor. It’s all ‘send a photograph’ and like we’re talking right now. Again, you can’t see my hands. And a lot of a diagnosis of anxiety, you can tell by the hands. So, to me, the GPs don’t necessarily get a true picture. “ ID2, aged 60–69, single, no children, living alone.*

Remote consultations created a barrier for support professionals to be able to pick up on nuances when participants were trying to communicate that their partner they were living with was being abusive, as one participant described of her interactions with a health visitor:

*“In terms of support for me, I felt like a lot of people didn’t pick up on what I was going through because I didn’t have that face-to-face contact with people. I can remember talking to a health visitor about my partner shouting at my baby, and she was like, oh, yes, men can suffer with post-natal mental health as well.” ID12, aged 30–39, single, parent, living with children.*

When participants did interact with services in-person, and tried to indirectly get the attention of those providing care, conversations about safeguarding issues were perceived as inadequate or avoided, as one participant explained regarding her contact with a community prescriber:

*“I think, because it was lockdown, I don’t think there was (pause) Almost, even professionals, in their heads, were making more excuses and being like, oh, well, what was normal isn’t normal anymore, so… You know? I think there’s a number of people who I probably hinted at stuff to who didn’t do anything, or missed what I was saying, or didn’t think about it in the right way. Yes.” ID12, aged 30–39, single, parent, living with children.*

Some participants that were interacting with formal services described feeling as though they were receiving a low standard of care because the behaviour that they needed to change was not being addressed:

*“We can talk all beautiful things about how we think the relationship should be and it all looks nice, but then the actual behaviour which is abusive was not addressed at all. Nobody hears that. What is the point of the social service meeting at all if that is not addressing the behaviour which would happen?” ID9, aged 20–29, separated, parent, living in temporary accommodation.*

##### Some support options disappeared suddenly during the pandemic

Several participants described an escalation of abuse alongside a complete withdrawal of formal support. Contacting a service was often met with the response that they were unable to help: *“Contacting anybody they would say ‘oh, we are overwhelmed, we can’t provide too much support.’” ID9.* Although some abuse support group meetings proceeded remotely, this was not universal, leaving some participants with support options that disappeared suddenly. For those unable to access online support groups, one of the most important losses was that of discussing shared experiences with other peers:

*“It was really difficult. I used to go to… domestic violence groups, and meet other women… And we used to have breakfast clubs in the morning… And I loved it. It was the place where I could actually meet other women like me… And then it all went overnight.” ID14, aged 20–29, single, no children, living in temporary accommodation.*

Some participants felt that the reduction in support was due to Violence Against Women and Girls (VAWG) professionals facing higher workloads and increased responsibilities in their personal lives associated with the pandemic*.* One of the consequences of support services being overwhelmed or unable to operate during the pandemic was that some participants disengaged altogether from services that they were previously in touch with. Some even stopped asking for help:

*“In the second or third lockdown, I didn’t engage with them (support services) anymore, because I knew there was nothing they could do. So, what was the point? Everywhere was shut. I still felt like a sitting duck.” ID14, aged 20–29, single, no children, living in temporary accommodation.*

##### Friends and family unable to help because they were following COVID rules

Support from neighbours, friends and family was restricted because many of those who provided help in pre-pandemic times were concerned about their own COVID-19 risk and upholding the rules. This was particularly so when social contacts were considered clinically extremely vulnerable:

*“Our older neighbours were left really fearful, and suddenly, all of their support went, so I was running round after them as well, make sure they were okay. Yes, it was pretty bad, really, pretty scary times.” ID7, aged 50–59, divorced, parent, living alone.*

As one participant explained, this meant having to live in an abusive situation for longer than they would have wanted:

*“I asked [my ex-husband] to find somewhere to go. And he phoned round all his friends, and nobody would take him because of the restrictions, we’ve got friends who were really sticking to everything, as we were, everyone had come so far, nobody wanted to breach it, so there was nowhere he could go.” ID5, aged 40–49, divorced, parent, living with children.*

Some felt that they did not want to bother their social networks for emotional or practical support because they perceived the weight of domestic violence (DV) and the pandemic on others to be too “immense.” For one participant, the shift in social dynamics and perceived lack of privacy due to families being together at home all the time meant she was unable to approach her friends living with their families for support.

*“All my family were having their own struggles, all my friends were mutual friends with my husband, I couldn’t turn to any of them, and my one friend in [Location], was locked down with her [family], so she couldn’t even speak privately. So, I just went from one day to the next… waiting it out.” ID7, aged 50–59, divorced, parent, living alone.*

Other participants said that neighbours around them knew of the abuse occurring during lockdown but did not intervene, perhaps because of the lockdown situation:

*“We had neighbours both sides… They must have heard all sorts, and yet they never did anything…. And I do wonder, if we weren’t in a lockdown, whether they would’ve thought that behaviour less acceptable and actually thought this isn’t normal. But I think, probably because we were in lockdown, I think a lot of people justified, ‘oh, people are going to be more shouting at each other’.” ID5, aged 40–49, divorced, parent, living with children.*

Some participants pointed out that their experiences of long-standing gender bias and homophobia in their community were more pronounced during the lockdown. For example, as one participant described, her neighbours witnessed IPV from a woman towards another woman, but they did not acknowledge it or intervene, leading to further feelings of exclusion and isolation from her local community.

*“When I was reading about all these or was going online and hearing about all these idyllic streets of people, around the country, looking after their neighbours. And not one neighbour on my street asked if I was okay… And I thought, at the time, this, again, it shows people like me where we don’t stand in the community, because you’d think, during a pandemic, there would be something.” ID8, aged 50–59, single, no children, living alone.*

#### Increased feelings of fear, isolation, and loss of control

Although many participants experienced an improvement in aspects of their social lives during the pandemic, there were still points in the pandemic where a decline in mental health and wellbeing was more pronounced than others. For some participants, the start of the pandemic was the most difficult time due to pressurised relations in the family home, but for others living alone, it was the extended periods of time spent in lockdown without formal and informal support that was attributed to a decline in their mental health.

##### Feelings of fear and isolation that collided with the pandemic and abuse

Most participants described feelings of loneliness and isolation throughout their pandemic experience. Some attributed these feelings to different seasons or waves of the pandemic and an increase in severity of social distancing restrictions. Feelings of loneliness were often attributed to being alone after leaving an abusive relationship during the pandemic: *“other than him, I wasn’t seeing anybody”* and therefore, the ability to engage in any post-break up rituals was restricted. Other participants described feeling lonely because the abuse had taken up so much of their time, thinking space and energy.

*“I think I did have this empty space, when my ex left. Empty, like I’d spent a lot of time worrying about this relationship, for the past five years, and expending a lot of energy on it… a fourth of my brain was probably engaged in dealing with that situation, and now that situation was gone. And then, [Location] locked down, I’m here, I have this empty space, what do I do with it? And I didn’t know how to deal with it… So, that was my sort of thing, and then this whole I’d left this abusive relationship, and the post-abuse situation… But mine exactly coincided with the pandemic.” ID19, aged 30-39, divorced, parent, living with children*

Feelings of loneliness were often pronounced for those who lived alone, were not working, had recently moved, or lived in a rural area. For instance, this was depicted by one participant describing feeling additional layers of loneliness during the pandemic after ending her relationship with an ex-partner.

*“I felt that even though there are people there, it’s not the same as having somebody in the house, next door, down the road. That was the hardest part… I’ve lost a lot coming out of this. She hasn’t lost anything, because she’s living with her friend. I’ve said, I don’t have that cushion.” ID8, aged 50–59, no children, living alone.*

##### The pandemic took away certainty and control

Participants who had experienced mental health difficulties prior to the pandemic, described feeling that the certainty and control that they had previously gained was taken away.

*“I think one of the most difficult things when you have experienced domestic abuse is your life is out of your control. So, somebody else is trying to take control. And the more you try and take control back, it actually becomes more frightening because the abuser gets worse… So, with the lockdown, a pandemic situation, I was here. I have no idea where he was. Everybody has to be at home. That doesn't mean to say he was. He’s a criminal, for heaven's sake… and it's that unknown. The unknown is very frightening.” ID2, aged 60–69, single, no children, living alone.*

For some participants, one consequence of not feeling in control was the adoption of riskier behaviours, such as undertaking sex work that conflicted with their boundaries or entering situations with strangers that they knew posed dangers during the pandemic. Others said they sought out harmful relationships that they knew were unhealthy, as a salve for their emotional wellbeing and to help cope with loneliness.

*“I was in such a vulnerable place, without realising it, that I was just pleased to have that kind of opportunity to have a relationship with somebody that wasn’t two feet away from them. Literally, when I look at it now, it really makes sense, because I think if it had been last year, I wouldn’t have done what I did. I think that that’s what it was, I felt vulnerable and alone, and as people say, sometimes negative attention’s better than none.” ID8, aged 50–59, single, no children, living alone.*

##### Fear about risks from domestic abuse felt far worse than fear of being exposed to COVID-19

In the context of escalating violence and abuse at home, some participants said that their fears about risk of contracting COVID-19 "paled in comparison" to harmful consequences from abuse. There were parallels drawn between “life in lockdown” and a life of living with some forms of abuse, both of which felt like a “prison” to some participants.

*“…I think because of what I’d been through with my ex-partner being so horrible, I was like, oh, what’s a pandemic in relation to that, it really didn’t feel as scary or significant as the other stuff that was going on.” ID20, aged 30–39, parent, living with children.*

As one participant explained, this meant that her experience of social distancing restrictions during the first lockdown were not dissimilar to her everyday life before the pandemic.

*“When we went into the first lockdown, I was still strong. People were going, ‘oh God, this is awful.’ I just said, ‘welcome to my world.’ This is what I've lived like… Locked in, not seeing anybody, keeping my distance… I have self-isolated [for years] to be safe, mentally, for my own self.” ID2, aged 60–69, single, no children, living alone.*

#### Some forms of support were more readily available and accessible during the pandemic

Although a majority of participants reported difficulties with accessing formal and informal support, a smaller group said that their perceived levels of support were higher during the pandemic. At a time where many access routes to support had been cut off, being able to engage with self-help became of utmost importance.

##### Increased access to formal support during the pandemic

Although most participants found it difficult to access services during the pandemic and found services to be unsatisfactory compared with pre-pandemic face-to-face support, some acknowledged that remote service provision had benefits, e.g., rapid responses, around the clock availability, and easy access:

*“The domestic abuse support from the domestic abuse police officer, DAO, used to be face to face, and it is telephone now only, which is definitely better.” ID2, aged 60–69, single, no children, living alone.*

Some participants who were previously supported by service providers pre-pandemic experienced a continuation of support, which proved to be helpful: *“So I was already in both DV support and counselling before the pandemic, but then it moved online.”* Some services, whilst remote, did not disappear entirely which meant that for those with more complex needs there was still someone to turn to via telephone or email. Having access to these services was essential, particularly for women who needed help with accessing food, benefits, and housing:

*“Yes, it was the [redacted] support service. They were the ones that got me into the hostel to begin with. And they were the ones who really pushed the police to move me. And she (the support worker) was the one that would ring me up every day and work out housing… options for me and stuff like that. I couldn’t have done it without her.”* ID14, aged 20–29, *single*, no children, living in temporary accommodation.

##### Support needs being met through comprehensive and innovative interventions

Although many participants described situations during the pandemic where their support needs were not being met, there were some cases where support was sought and provided in a meaningful, timely and helpful fashion. As one participant described, she was referred to an organisation who provided links with a range of services that she needed at the time:

*“They referred me into the organisation that covers my area, and they did a risk assessment based on the abuse. And they were like, oh, you’re still quite high, you’ll get a support worker, so I got a support worker. And they were like, okay, let’s get you a solicitor…they [also] run a weekly programme for women who’ve been through abuse, which basically looks at identifying different types of abuse, I guess, and also then looks at what’s healthy. So, that’s been quite helpful, but then they also just ring for a check-in.” ID12, aged 30–39, single, parent, living with children.*

For those participants feeling overwhelmed with what was happening in their lives in the context of the pandemic, receiving clear, decisive support was essential:

*“And this person, God bless her, she was like, no, this is a really dangerous situation, and she completely understood what I was going through, and she was like, no, you’re not going through, forget about your thing, so basically make sure that I got housing. So, I got housing by, I think, 4 PM…. the next morning…Sometimes you just need actual, substantive support, [like] housing. Someone to do the research for you, maybe, if you’re feeling too overwhelmed, or a GP that understands, and is like, okay, maybe you have this problem, and maybe this is a solution, and is not dismissive.” ID19, aged 30–39, divorced, parent, living with children.*

Some participants described new ways of accessing support during the pandemic. Social media provided an outlet to talk about DA with existing social contacts, share information about DA support, and join groups for social support.

*“On social media, a couple of friends who didn’t know what was going on would signpost people to places, websites and stuff, and that felt very supportive. So I think social media could play a part in adverts, I suppose, saying if you need help, here it is, because you can just see that without having to go searching for it.” ID5, aged 40–49, divorced, parent, living with children.*

Several women posted in online Facebook groups for mums, about their experiences for peer support. In other cases, women were able to access support and information from chance encounters in the community, such as finding a flyer in a local park with abuse support helpline details, an option to report abuse at the local pharmacy (also known as “Ask for ANI”), or access to a safe room at a supermarket shopping centre:

*“When I was in [the supermarket], when I had this incident, those staff were exceptional. They said, we've got a safe room. I just had to say, can you look after my trolley? I’ve just got to leave. They said, are you all right? No, I have a stalker, domestic abuser, and he’s here. They said, don’t worry, we’ll take this, come into our room. And it was their staff room, but they class it as a safe room.” ID2, aged 60–69, single, no children, living alone.*

##### Engaged more with self-help and self-care during the lockdown

A number of participants used physical activities such as running, walking and home exercise to boost their mood. Living in lockdown, after escaping abuse, meant that there was more time for self-care and prioritising mental health and wellbeing.

*“Before, I didn’t look after myself. Well, I did, but I didn’t prioritise my own self-care. So, prioritising the children, working, obviously in the abusive relationship I was made to prioritise him, which thankfully is not an issue anymore. But the pandemic has made me view the self-care thing better.” ID13, aged 30–39, single, parent, living with children.*

Most participants used mental relaxation techniques (e.g., meditation and mindfulness), gardening, volunteering, house organising and decorating, reading, art and cooking to help cope with their experiences of lockdown and abuse. *“I tried different self-help books, and I’ve got one focusing on moving on after domestic abuse.” ID10.* Some participants used writing to help process their experiences to “self-heal” from experiences of abuse: *“I wrote down these things that I was going to tell myself every day.” ID3.* Participants reported appreciating the “small things” to distract themselves from hardships. Some already used these tools before the pandemic, whilst others discovered them during lockdown.

*“It’s doing mindful things, where your brain is not distracted by the unwanted thoughts… Appreciating sunshine and the weather. I keep a positive diary… So, every day, I ended up putting positive things in it… So, that was good. And again, these are things which I suppose I've carried through from before.” ID2, aged 60–69, single, no children, living alone.*

#### Psychosocial wellbeing underwent a transformation during the pandemic

In the absence of ongoing abuse, later lockdowns provided a protective space for some participants to take back control of certain aspects of their lives, leading to feelings of freedom, which had not been experienced for some time. Identity changes were particularly noted by participants with caregiving responsibilities, as many were navigating parenthood without an abusive partner in their home for the first time. Meanwhile, some described a new sense of purpose arising from their experience of the pandemic, which coincided with the ending of an abusive relationship and lifting of lockdown measures.

##### Reconnecting with sense of self after abuse ended

The ending of abuse amidst a global pandemic, meant that participants were exploring a new sense of identity outside of their abusive relationship. Many felt better able to reconnect with themselves due to extra time during lockdown coupled with the ending of an abusive relationship.

*“Living with him was very draining, I had to do a lot of work because he was incapable of being an adult by himself. So, I’ve been able to focus more on- (pause) Focus is the wrong word, just be myself more with less distractions.” ID20, aged 30–39, parent, living with children.*

For those who had been living with abuse for a long time, this was the first time that they had experienced single life or experimented with their sexuality. Having the combined experience of surviving abuse and living through lockdown, brought with it a renewed perspective about how participants said they wanted to live their life in the future. For example, some participants returned to work for the first time in a long time, re-evaluated their diet, or changed their spending habits. Some participants had been in abusive relationships for many decades before deciding to leave during the pandemic. They described feeling a renewed sense of resilience and independence coming out of the pandemic:

*“I’ve made it through that year of being on my own, in my own place, and I’ve made it through the seven months before that, of horrific pressure.” ID7, aged 50–59, divorced, parent, living alone*

For many who had been confined to their homes during lockdown and simultaneously experiencing abuse, the lifting of lockdown brought with it an extraordinary sense of freedom and confidence.

*“I think the word that for me characterises my learning from this experience is freedom. And I think freedom can be in lockdown because it’s my freedom to do what I want. Even though within the limitation that I’m allowed to, but I’m still free to do what I want as a person.” ID1, aged 20–29, single, no children, living with housemates.*

Several participants found it a challenge to manage how they felt about their changing identity, whilst also navigating how their young children felt about the pandemic and abusive parent leaving the family home. As one participant described, she was trying to process how she felt about being a lone parent without an abusive partner, whilst supporting her child, without the usual supports of friends and family due to the pandemic.

*“I definitely felt like, just as one adult and one child, it was worse, because it wasn’t like I was all alone. I couldn’t completely zone out, as a mum, I had to be zoned in…I couldn’t complete, because of the situation, because of having my son at home all the time. And my son, I think he was feeling, his dad had left, his friends had disappeared, his teachers, that he loved so much, were cut out, and he really needed a lot of attention and a lot of one-on-one contact, and he was really feeling isolated.” ID19, aged 30-39, divorced, parent, living with children*

##### Socialising more now for the first time in a long time

In the aftermath of long-term IPV, the social worlds of many participants had condensed, through lost friendships, and through lives being controlled. Many of the participants indicated that the combination of feeling freed from abuse and the easing of social distancing restrictions was coupled with a reacquaintance with socialising again. Changes in socialising were facilitated by online access. Some were able to engage with group activities, like online quizzes, that they previously had not been to before. The shift to online socialising was particularly valued by participants with caregiving responsibilities, whereby, they could attend events they previously would not have been able to.

*“The positive things were the online shift whereas I could attend some of the social events I wouldn’t otherwise be able to attend with a little child. So, if it’s after their sleep or I could sort of manage with her playing by my side and attending an online event. So, that was a good thing where before if it wasn’t online, I couldn’t have access to this.” ID9, aged 20–29, separated, parent, living in temporary accommodation.*

Some participants described a lack of confidence about making new friendships prior to the pandemic, but having a collective experience of being in the lockdown made relating to others feel less intimidating:

*“I try to have better connections with people… I don’t know if it’s made it easier to do that in the pandemic because other people are doing that as well. It is something being freeing; we just sort of… I don’t know, I feel like it’s easier to talk to strangers now because everyone again has been in the same boat, we all have something in common.” ID20, aged 30–39, parent, living with children.*

## Discussion

In this study, we investigated the impact of the COVID-19 pandemic on the lives of female IPV survivors, including how access to services and support was affected during lockdown restrictions. As reflected in international research, the participants we interviewed described pandemic-related tensions within their family homes [45], including increased financial strain and childcare responsibilities [46]. Participants described how their usual routes of escape from abuse had been removed due to lockdown measures, and this was linked to an increase in the frequency and severity of abuse. We identified a number of potential policy implications arising from this research which we describe below, including the need for trauma-informed guidelines in the event of future social distancing restrictions, prioritising availability of community-based intervention points for victims to report abuse, and ensuring self-help resources are continuously available for survivors when formal or face-to-face support options are restricted.

### Exploitation of pandemic-related changes to perpetuate abuse

The COVID-19 pandemic not only changed the daily lives of people being subjected to violence and abuse, but also the nature of crimes taking place [47, 48]. Public narratives about the dangers of abuse during the pandemic have focused on the risks of physical harms with little emphasis on coercive control and emotional forms of abuse [49]. Our research adds to this body of knowledge by highlighting how the nature of non-physical forms of abuse evolved during the pandemic. Participants reported new manifestations of coercive control and other harmful behaviours during the COVID-19 pandemic. This included having basic needs restricted by perpetrators, with no means to leave the house for supplies, over-interpretation of the rules (resulting in being involuntarily confined to the house for months at a time), perpetrators disguising their identity with personal protective equipment (PPE), and those who were extremely clinically vulnerable being deliberately exposed to the virus. International evidence suggests that prevalence of coercive control rose during COVID-19 lockdowns and this has highlighted how ineffective many pre-existing policies are in supporting those requiring protection from it [49]. It is essential that future pandemic restrictions are developed with consideration that instances of coercive control are likely to rise, with abusive individuals seeking to find new ways to exert harm on others.

### Communication of clear public health messaging combined with a national abuse safety plan

In March 2020, the UK Home Secretary Priti Patel said that "Whilst our advice is to stay at home, anyone who is at risk of, or experiencing, DA, is still able to leave and seek refuge. Refuges remain open, and the police will provide support to all individuals who are being abused—whether physically, emotionally, or otherwise.” [50]. However, the women in our study described why leaving was not always an option to them, with reasons such as having a long-term illness that pre-dated the pandemic or caring for a child that was clinically vulnerable. Other reasons directly caused by the pandemic included having nowhere to go if they were to leave home due to the abuse or feeling compelled to stay in the family home with an abusive spouse for the perceived welfare of young or grown children who moved home during the lockdown periods. Despite some shelters remaining open during the lockdown periods, many were unable to take on new clients [51] because they were already facing pre-existing challenges from funding cuts and the long-term impacts of austerity [30]. So, while public awareness campaigns and announcements for victims of abuse are crucially needed in a pandemic [52], these efforts in the future must be also part of a broader plan to address the barriers that already prevent victims from being able to leave, which are likely to be exacerbated during pandemics and emergencies. Services like refuges must be sufficiently funded and provisioned in the first place to ensure they are able to meet the needs of those who can “leave and seek refuge” from abuse.

### Ensuring access to community-based intervention points are continuously available

The government made several specific announcements during the first year of the pandemic regarding support for victims and survivors of abuse, including a contribution of £1.6 billion to local councils for support services. Part of the government’s response to abuse concerns during the pandemic included partnering with UK pharmacies in January 2021, with an initiative called “Ask for ANI (Action Needed Immediately)” to help improve access points for victims to disclose abuse [53]. Perspectives shared by our participants provide support for these intervention points, and that multiple initiatives are essential for reaching people experiencing abuse, irrespective of the presence of a pandemic. Whilst local government and third-sector services are well aware of the need to tackle abuse in the community, there are still barriers that prevent their efforts from reaching victims and survivors [54]. Frontline workers, including delivery drivers, post office workers and contractors may be more likely to recognise and be able to intercept when someone is showing signs of needing help with abuse [55]. Therefore, specialised training is recommended for these customer-facing professions to equip staff with tools to appropriately report their concerns to those who can intervene [54, 55].

### Addressing problems with abuse risk assessment during remote service provision

Evidence suggests that some abuse services were underutilised during lockdown conditions, compared to pre-pandemic times [31, 56]. Our results indicate reduced service use was not due to lack of demand but an increase in controlling behaviours and risk of being overheard by an abusive partner at home. Most participants in our study experienced difficulties with remote service provision when accessing support for their health, safety and wellbeing during social distancing restrictions. Many described problems with building rapport and identified safety risks from being able to hide emotions or physical signs of abuse when being seen only by video or telephone. Domestic violence practitioners have voiced similar concerns regarding remote risk assessment, with some services being directed to use the same assessment tools as pre-COVID times [57], which may not have been suited for use during COVID restrictions. Research involving social workers highlights how some services may not have been equipped with the right training or access to resources that would have enabled identification of safeguarding concerns during the pandemic, leaving some practitioners feeling alone and unsupported [58]. Consequently, more work is needed to address these problems in remote risk assessment in order to prepare for future pandemics and disasters. This might be addressed through development of best practice guidelines for risk assessment, regular monitoring of changes in service effectiveness, and involving people with lived experience throughout service adaptation planning [57].

### Provision of self-help resources in times when service provision is adversely affected

Pandemic-related changes meant that for many victims and survivors of abuse, formal and informal support was harder to access than ever, including cases where services shut down completely or when travel restrictions were in place [46]. Barriers in access to support in times of crisis highlights the importance of self-help options for people trying to regulate their mental health and wellbeing. Ensuring that self-help resources are available and promoted in times of future pandemics and disasters can help further support many different groups including those who have been subjected to violence and abuse. All participants we interviewed engaged in coping techniques to protect their mental health, including use of mindfulness meditation and distraction activities, which have been shown to decrease depression, anxiety and PTSD symptoms in abuse victims and survivors [59, 60]. Another positive coping strategy utilised by participants in this study was volunteering, known to be associated with improved affect and sense of purpose [61]. For future pandemics, it is recommended that additional public health campaigns are developed to promote the benefits of these coping techniques to improve self-help at home where service provision is restricted.

### Strengths and limitations

A strength of the study is that we were able to conduct rich and detailed interviews with a range of women including within the context of straight and queer relationships, with men, women and people identifying as non-binary. Although we did not ask participants to state their socioeconomic status, there is evidence of a spectrum of socioeconomic positions among participants, including some describing financial hardship and temporary housing that intersected with their experiences of other difficulties throughout the pandemic. Though rich and novel data have been obtained, the study had some inherent limitations. Amidst an ongoing pandemic, the UK had been in one national lockdown by the time we conceived this study plan, but the published literature on the gravity of consequences from intimate partner violence was sparse. We undertook this research with great care and caution for the safety of research participants, and only interviewed women who were safe from abuse at the time of interview. We, therefore, are unlikely to have generated themes that fully resonate with the experiences of all people who were subjected to ongoing IPV during the COVID-19 pandemic. Although we interviewed women with diverse experiences and relationships, the sample is homogeneous in terms of educational attainment and ethnicity, consisting of predominantly White women (72%) with postgraduate education (56%). People from ethnic minority groups are disproportionately represented in DA statistics [2] and this reflects an important perspective that is missing in our research. Although we did not examine this topic directly in our research, evidence suggests that instances of child abuse and child witnessed IPV may have risen during the pandemic [62, 63] and this also remains an important avenue of future enquiry.

## Conclusions

During a time of unprecedented circumstances, the COVID-19 pandemic highlighted existing barriers that abuse victims and survivors are presented with when trying to access support, and these issues only deepened during the crisis that COVID-19 posed. Third-sector services, health providers, researchers and policymakers must now plan for the after-effects of the COVID-19 pandemic, where many abuse victims and survivors faced support restrictions in the UK for considerable lengths of time. There is a pressing need to improve safety plans for supporting victims in global emergencies and pandemics. Our results highlight the importance of multifaceted intervention points and awareness campaigns so that victims can reach out and feel supported in the midst of a pandemic. This includes having community-based hubs for victims to disclose abuse and online platforms for receiving information and peer support. Participants in our study described adopting a form of survival mode to cope with abuse and the pandemic at the same time, which highlights the importance of information and offers of support for people who left abusive relationships during the pandemic, long after the threat of COVID-19 has ended.

## Supplementary Information

**Additional file 1:** Brief overview of lockdown restrictions in England that affected participants in the study.

**Additional file 2:** Participant screening form.

**Additional file 3:** Interview guide.

**Additional file 4:** Examples of full quotes from research participants.

## Data Availability

The datasets generated and/or analysed during the current study are not publicly available due to this containing information that might compromise the identity of research participants, and these data are not available from the corresponding author on request.

## References

[1] World Health Organisation. Coronavirus disease 2019 (COVID-19) Situation Report – 51. 2020. Available from: https://www.who.int/docs/default-source/coronaviruse/situation-reports/20200311-sitrep-51-covid-19.pdf?sfvrsn=1ba62e57_10.

[2] Chandan JS, Gokhale KM, Bradbury-Jones C, Nirantharakumar K, Bandyopadhyay S, Taylor J (2020). Exploration of trends in the incidence and prevalence of childhood maltreatment and domestic abuse recording in UK primary care: a retrospective cohort study using ‘the health improvement network’ database. BMJ Open.

[3] Carney M, Buttell F, Dutton D (2007). Women who perpetrate intimate partner violence: a review of the literature with recommendations for treatment. Aggress Violent Behav.

[4] Finneran C, Stephenson R (2013). Intimate partner violence among men who have sex with men: a systematic review. Trauma Violence Abuse.

[5] Home Office. The economic and social costs of domestic abuse. 2019. Available from: https://www.gov.uk/government/publications/the-economic-and-social-costs-of-domestic-abuse.

[6] Coker AL, Davis KE, Arias I, Desai S, Sanderson M, Brandt HM (2002). Physical and mental health effects of intimate partner violence for men and women. Am J Prev Med.

[7] Ansara DL, Hindin MJ (2011). Psychosocial consequences of intimate partner violence for women and Men in Canada. J Interpers Violence.

[8] Olsen W, Gash V, Sook K, Zhang M (2018). The gender pay gap in the UK: evidence from the UKHLS (DFE-RR804).

[9] Stanfors M, Jacobs JC, Neilson J (2019). Caregiving time costs and trade-offs: gender differences in Sweden, the UK, and Canada. SSM Popul Health.

[10] Xue B, McMunn A. Gender differences in unpaid care work and psychological distress in the UK Covid-19 lockdown. Tran TD, editor. PLoS One. 2021;16(3):e0247959. 10.1371/journal.pone.0247959 PMC7932161 33662014

[11] Paul E, Razai MS, Burgess RA, Burton A, Fancourt D, McKinlay AR. Experiences of different types of discrimination in the UK during the first 16 months of the COVID-19 pandemic: A mixed methods study. SocArXiv. 2022. Available from: https://osf.io/bjwg3. [Cited 27 Nov 2022].

[12] Cleaver K, Maras P, Oram C, McCallum K (2019). A review of UK based multi-agency approaches to early intervention in domestic abuse: Lessons to be learnt from existing evaluation studies. Aggress Violent Behav.

[13] Mapayi B, Makanjuola ROA, Mosaku SK, Adewuya OA, Afolabi O, Aloba OO (2013). Impact of intimate partner violence on anxiety and depression amongst women in Ile-Ife Nigeria. Arch Womens Ment Health.

[14] Rezaeian M (2013). The association between natural disasters and violence: a systematic review of the literature and a call for more epidemiological studies. J Res Med Sci Off J Isfahan Univ Med Sci.

[15] Xu J, Wang Z, Shen F, Ouyang C, Tu Y (2016). Natural disasters and social conflict: a systematic literature review. Int J Disaster Risk Reduct.

[16] John N, Casey SE, Carino G, McGovern T (2020). Lessons never learned: crisis and gender-based violence. Dev World Bioeth.

[17] Fisher S (2010). Violence against women and natural disasters: findings from post-tsunami Sri Lanka. Violence Women.

[18] Schumacher JA, Coffey SF, Norris FH, Tracy M, Clements K, Galea S (2010). Intimate partner violence and hurricane katrina: predictors and associated mental health outcomes. Violence Vict.

[19] World Health Organisation. Interpersonal violence & disasters. 2005a. Available from: https://www.who.int/violence_injury_prevention/publications/violence/violence_disasters.pdf.

[20] Agüero JM (2021). COVID-19 and the rise of intimate partner violence. World Dev.

[21] Gosangi B, Park H, Thomas R, Gujrathi R, Bay CP, Raja AS (2021). Exacerbation of physical intimate partner violence during COVID-19 pandemic. Radiology.

[22] Moore G, Buckley K, Howarth E, Burn AM, Copeland L, Evans R (2022). Police referrals for domestic abuse before and during the first COVID-19 lockdown: an analysis of routine data from one specialist service in South Wales. J Public Health.

[23] Sabri B, Hartley M, Saha J, Murray S, Glass N, Campbell JC (2020). Effect of COVID-19 pandemic on women’s health and safety: a study of immigrant survivors of intimate partner violence. Health Care Women Int.

[24] Bradbury-Jones C, Isham L (2020). The pandemic paradox: the consequences of COVID-19 on domestic violence. J Clin Nurs.

[25] Kelly J, Morgan T. Coronavirus: Domestic abuse calls up 25% since lockdown, charity says. BBC News. 2020. Available from: https://www.bbc.co.uk/news/uk-52157620.

[26] Barbara G, Facchin F, Micci L, Rendiniello M, Giulini P, Cattaneo C (2020). COVID-19, lockdown, and intimate partner violence: some data from an Italian service and suggestions for future approaches. J Womens Health.

[27] Wright EN, Miyamoto S, Richardson C (2021). The impact of COVID-19 restrictions on victim advocacy agency utilization across Pennsylvania. J Fam Violence.

[28] Bybee DI, Sullivan CM (2002). The process through which an advocacy intervention resulted in positive change for battered women over time. Am J Community Psychol.

[29] Human Rights Watch. UK Failing Domestic Abuse Victims in Pandemic. 2020. Available from: https://www.hrw.org/news/2020/06/08/uk-failing-domestic-abuse-victims-pandemic. [Cited 29 Sep 2021].

[30] Dawsey-Hewitt S, Jnagel T, Kalia S, Royal K, Seshadri S, Sutherland L, et al. Shadow pandemic - shining a light on domestic abuse during covid. Women’s Aid Federation of England; 2021. Available from: https://www.womensaid.org.uk/wp-content/uploads/2021/11/Shadow_Pandemic_Report_FINAL.pdf.

[31] Lyons M, Brewer G (2022). Experiences of intimate partner violence during lockdown and the COVID-19 pandemic. J Fam Violence.

[32] Ravi KE, Rai A, Schrag RV (2022). Survivors’ experiences of intimate partner violence and shelter utilization during COVID-19. J Fam Violence.

[33] Mahapatro M, Prasad MM, Singh SP (2021). Role of social support in women facing domestic violence during lockdown of Covid-19 while cohabiting with the abusers: analysis of cases registered with the family counseling Centre, Alwar India. J Fam Issues.

[34] Weiland N, Smith M, Sullivan ES. Covid-19: Britain Begins to Reopen, Emerging From One of the World’s Longest Lockdowns. The New York Times. 2021. Available from: https://www.nytimes.com/live/2021/04/12/world/covid-vaccine-coronavirus-cases? [Cited 21 Oct 2021].

[35] Wright L, Fancourt D, Bu F. COVID-19 Social Study User Guide. OSFHome. 2021. Available from: https://osf.io/jm8ra/. [Cited 13 Jan 2022]

[36] Evans ML, Lindauer M, Farrell ME (2020). A pandemic within a pandemic — intimate partner violence during Covid-19. N Engl J Med.

[37] Braun V, Clarke V (2021). Can I use TA? Should I use TA? Should I *not* use TA? Comparing reflexive thematic analysis and other pattern-based qualitative analytic approaches. Couns Psychother Res.

[38] Braun V, Clarke V, Hayfield N (2022). ‘A starting point for your journey, not a map’: Nikki Hayfield in conversation with Virginia Braun and Victoria Clarke about thematic analysis. Qual Res Psychol.

[39] Braun V, Clarke V (2006). Using thematic analysis in psychology. Qual Res Psychol.

[40] Braun V, Clarke V. Thematic analysis | a reflexive approach. n.d. Available from: https://www.psych.auckland.ac.nz/en/about/thematic-analysis.html. [Cited 12 Feb 2022].

[41] Longino HE. Feminist Epistemology. In: Greco J, Sosa E, editors. The Blackwell Guide to Epistemology. Oxford, UK: Blackwell Publishing Ltd; 2017. p. 325–53. Available from: https://onlinelibrary.wiley.com/. 10.1002/9781405164863.ch14. [Cited 3 Mar 2022].

[42] Terry G, Hayfield N, Clarke V, Braun V (2017). Thematic analysis. The SAGE Handbook of Qualitative Research in Psychology.

[43] QSR International. Nvivo. 2021. Available from: https://www.qsrinternational.com/nvivo-qualitative-data-analysis-software/home.

[44] Byrne D. A worked example of Braun and Clarke’s approach to reflexive thematic analysis. Qual Quant. 2021. 10.1007/s11135-021-01182-y. [Cited 21 Mar 2022].

[45] McNeil A, Hicks L, Yalcinoz-Ucan B, Browne DT. Prevalence & correlates of intimate partner violence During COVID-19: a rapid review. J Fam Violence. 2022. 10.1007/s10896-022-00386-6. [Cited 6 May 2022]. 10.1007/s10896-022-00386-6 PMC8961087 35368512

[46] Michaelsen S, Djiofack H, Nombro E, Ferlatte O, Vissandjée B, Zarowsky C (2022). Service provider perspectives on how COVID-19 and pandemic restrictions have affected intimate partner and sexual violence survivors in Canada: a qualitative study. BMC Womens Health.

[47] Regalado J, Timmer A, Jawaid A (2022). Crime and deviance during the COVID-19 pandemic. Sociol Compass.

[48] Gregory A, Williamson E (2021). ‘I think it just made everything very much more intense’: a qualitative secondary analysis exploring the role of friends and family providing support to survivors of domestic abuse during the COVID-19 pandemic. J Fam Violence.

[49] Smyth C, Cullen P, Breckenridge J, Cortis N, Valentine K (2021). COVID-19 lockdowns, intimate partner violence and coercive control. Aust J Soc Issues.

[50] BBC News. Coronavirus: Domestic abuse victims “still allowed to leave home.” 2020; Available from: https://www.bbc.co.uk/news/uk-52081280.

[51] Jarnecke AM, Flanagan JC (2020). Staying safe during COVID-19: How a pandemic can escalate risk for intimate partner violence and what can be done to provide individuals with resources and support. Psychol Trauma Theory Res Pract Policy.

[52] Haag HL, Toccalino D, Estrella MJ, Moore A, Colantonio A (2022). The shadow pandemic: a qualitative exploration of the impacts of COVID-19 on service providers and women survivors of intimate partner violence and brain injury. J Head Trauma Rehabil.

[53] House of Commons. Domestic abuse and Covid-19: a year into the pandemic. 2021. Available from: https://commonslibrary.parliament.uk/domestic-abuse-and-covid-19-a-year-into-the-pandemic/.

[54] Rauhaus BM, Sibila D, Johnson AF (2020). Addressing the increase of domestic violence and abuse during the COVID-19 pandemic: a need for empathy, care, and social equity in collaborative planning and responses. Am Rev Public Adm.

[55] Campbell AM (2020). An increasing risk of family violence during the Covid-19 pandemic: strengthening community collaborations to save lives. Forensic Sci Int Rep.

[56] Bu F, Mak HW, Fancourt D (2021). Rates and predictors of uptake of mental health support during the COVID-19 pandemic: an analysis of 26,720 adults in the UK in lockdown. Soc Psychiatry Psychiatr Epidemiol.

[57] Cortis N, Smyth C, Valentine K, Breckenridge J, Cullen P (2021). Adapting service delivery during COVID-19: experiences of domestic violence practitioners. Br J Soc Work.

[58] Holt S, Elliffe R, Gregory S, Curry P. Social workers response to domestic violence and abuse during the COVID-19 pandemic. Br J Soc Work. 2022;19. 10.1093/bjsw/bcac119.

[59] Ghahari S, Khademolreza N, Poya FS, Ghasemnejad S, Gheitarani B, Pirmoradi MR (2016). Effectiveness of Mindfulness Techniques in Decreasing Anxiety and Depression in Women Victims of Spouse Abuse. Asian J Pharm Res Health Care.

[60] Hopwood TL, Schutte NS (2017). A meta-analytic investigation of the impact of mindfulness-based interventions on post traumatic stress. Clin Psychol Rev.

[61] Greenfield EA, Marks NF (2004). Formal volunteering as a protective factor for older adults’ psychological well-being. J Gerontol B Psychol Sci Soc Sci.

[62] Riddell CA, Neumann K, Santaularia NJ, Farkas K, Ahern J, Mason SM (2022). Excess Google Searches for Child Abuse and Intimate Partner Violence During the COVID-19 Pandemic: Infoveillance Approach. J Med Internet Res.

[63] Kovler ML, Ziegfeld S, Ryan LM, Goldstein MA, Gardner R, Garcia AV (2021). Increased proportion of physical child abuse injuries at a level I pediatric trauma center during the Covid-19 pandemic. Child Abuse Negl.

