# Supplementary material for: How did UK social distancing restrictions affect the lives of women experiencing intimate partner violence during the COVID-19 pandemic? A qualitative exploration of survivor views
Source: BMC Public Health. 2023 Jan 18;23:123. doi: 10.1186/s12889-023-14987-3 (PMC9845821; doi:10.1186/s12889-023-14987-3)
Supplement: Supplementary file 1 — Additional file 1: Brief overview of lockdown restrictions in England that affected participants in the study. [file 12889_2023_14987_MOESM1_ESM.docx]

Supplementary File 1 - Brief overview of lockdown restrictions in England that affected participants in the study

| Type of Restriction | Date range |
| --- | --- |
| “Shielding” guidance issued to people with serious pre-existing conditions such as chronic liver disease, sickle-cell disease and certain types of cancer (1,2) | 21 March 2020 |
| **First Nationwide UK lockdown announced** (3) – citizens required to stay at home to prevent spread of the virus | **23 March 2020** |
| Lockdown measures became legally enforceable (3) – citizens only allowed to leave home for essential reasons | 26 March 2020 |
| People allowed to leave home more than once per day for activities such as exercise and sunbathing (4) | 10 May 2020 |
| Schools reopened in phases (3) and people allowed to meet in groups of 6 outdoors (5) | 1 June 2020 |
| “Support bubble” initiative announced in England and Northern Ireland so that single person households can meet another household (6) | 13 June 2020 |
| “Nonessential” shops permitted to reopen (3) | 15 June 2020 |
| More lockdown restrictions lifted | 14 August 2020 |
| **Second lockdown in England announced** (3) | **31 October 2020** |
| Second lockdown ends in England (3), a tiered restriction system introduced (5) | 2 December 2020 |
| Tighter restrictions in London, Southeast England, and other parts of England where cases were rising due to Alpha variant (3) | 21-26 December 2020 |
| **Third lockdown in England enforced** (5) | **6 January 2021** |
| Stay at home order ends but some restrictions still in place (3) | 29 March 2021 |
| “Nonessential” shops and venues permitted to reopen (3) | 12 April 2021 |
| Most legal restrictions on social contact removed (3) | 19 July 2021 |
| New measures introduced to prevent spread of Omicron variant (3) | 8 December 2021 |

References

1. NHS England. Caring for people at highest clinical risk from COVID-19: Background and FAQs for patients. [Internet]. 2020. Available from: https://www.england.nhs.uk/coronavirus/wp-content/uploads/sites/52/2020/03/20200401-FAQs-Patients.pdf

2. Department of Health and Social Care, UK Health Security Agency. COVID-19: guidance on protecting people defined on medical grounds as extremely vulnerable [Internet]. 2020. Available from: https://www.gov.uk/government/publications/guidance-on-shielding-and-protecting-extremely-vulnerable-persons-from-covid-19

3. Institute for Government. Timeline of UK government coronavirus lockdowns and restrictions [Internet]. 2022. Available from: https://www.instituteforgovernment.org.uk/charts/uk-government-coronavirus-lockdowns

4. Gov.UK. Prime Minister’s statement on coronavirus (COVID-19): 10 May 2020 [Internet]. 2020. Available from: https://www.gov.uk/government/speeches/pm-address-to-the-nation-on-coronavirus-10-may-2020

5. Baker C, Kirk-Wade E, Brown J, Barber J. Coronavirus: A history of English lockdown laws [Internet]. House of Commons; 2021. Available from: https://commonslibrary.parliament.uk/research-briefings/cbp-9068/

6. BBC News. Coronavirus lockdown: “Support bubbles” begin in England and NI. 2020; Available from: https://www.bbc.co.uk/news/uk-53031844
