# Supplementary material for: How did UK social distancing restrictions affect the lives of women experiencing intimate partner violence during the COVID-19 pandemic? A qualitative exploration of survivor views
Source: BMC Public Health. 2023 Jan 18;23:123. doi: 10.1186/s12889-023-14987-3 (PMC9845821; doi:10.1186/s12889-023-14987-3)
Supplement: Supplementary file 2 — Additional file 2: Participant screening form. [file 12889_2023_14987_MOESM2_ESM.docx]

Supplementary File 2 – Participant Screening Form

**Screening questions**

Thank you for your interest in taking part in our study on Social Distancing during Covid-19 and the impact of COVID-19 on health and wellbeing

We need to collect some additional information to understand whether or not you are eligible to take part in the interview.

**What is your age? _______________________________**

**What is your gender? ____________________________**

Have you experienced any form of abuse from a former or current partner at any point during the current pandemic? (e.g since March 2020). This could be physical, emotional, sexual, financial abuse, or coercion, threats or intimidation.

Yes

No

Do you currently live with someone who is carrying out the abuse, either currently or in the past?

Yes

No

Are you currently at any risk of immediate harm or danger?*

Yes

No
