# Supplementary material for: How did UK social distancing restrictions affect the lives of women experiencing intimate partner violence during the COVID-19 pandemic? A qualitative exploration of survivor views
Source: BMC Public Health. 2023 Jan 18;23:123. doi: 10.1186/s12889-023-14987-3 (PMC9845821; doi:10.1186/s12889-023-14987-3)
Supplement: Supplementary file 4 — Additional file 4: Examples of full quotes from research participants. [file 12889_2023_14987_MOESM4_ESM.docx]

Supplementary file 4 – Examples of full quotes from research participants

| Theme | Subtheme | Quote Examples from Transcripts |
| --- | --- | --- |
| Stay at home orders meant being confined to a place where abuse was escalating | 1. Social distancing rules coincided with increasing relationship tensions | *“There was too much stress, and also the pandemic and then we also got ill with coronavirus. So, there was lots and lots of stress at the same time and accumulating financial stress. All that came out into how he interacted with me, how he interacted with the kids, becoming a bit more controlling, becoming a bit more particular about how things are done, with more arguments, more pressure and more control and all these things.” ID9*  *“Very quickly, my husband was drinking very heavily, staying up very, very late at night, and he was clearly, really struggling with the loss of his work, his identity, his income… He just found the whole pressure, he couldn’t communicate, at all, and what it did was highlight all the cracks that were there before the pandemic. Every little thing was suddenly magnified by the close proximity, by the restrictions, by the changes and everything else. Just everything fell apart, very, very quickly.” ID7* |
|  | 1. Decreased ability to avoid abuse during lockdown | *“I was less able to build things into my day, because he was here, in my face all the time, and if he needed to talk, then we talked and other things would take a back seat.” ID4*  *“Those cycles [of abuse] were happening so quickly, during the pandemic, it just really, really built up, and because I had no way of escaping it, no way of going to work and just relaxing, I was tense the whole time.” ID10*  *“The hardest thing was, during the pandemic… he knew I was at home. And he knew I couldn’t go anywhere. I couldn’t go out... And I felt like a sitting duck. And that was really difficult because I couldn’t then argue and get away. I couldn’t make excuses to go and meet someone, because everywhere was shut.” ID14*  *“As the lockdown extended, I became very trapped. I was trapped by the crime, and I’m still trapped... The pandemic doubled it really. Mainly because there was no escape.” ID2*  *“I knew she wouldn’t do anything drastic, violently, but I just wanted my own space, and I just didn’t feel that that was possible, and massively impacted by what’s been going on. There was just nowhere I could have gone, and I just didn’t feel there was any option to do anything. So, I’d kept having to put up with her screaming.” ID8*  *“We had a buyer, we put it on the market in November, and we had a buyer, very quickly, and it was meant to go in January. So, I thought I’ll stay here until it goes, and then that fell through, and then the abuse started again.” ID10*  *“And I told…my soon to be ex-husband, that this was going to end. We were going to end. But we needed to see the boys through... Just logistically, no matter how bad it was, I just couldn’t face shuffling around.” ID3* |
|  | 1. Manipulation and exploitation of social distancing rules by perpetrator to aid abuse | *“[social distancing rules were] all taken really literally, and it got to a thing where my partner was okay, you can’t actually leave the house without me, you can’t walk down the road to the shop on your own, I’m coming with you. We’re going or we’re not going. So, those rules and that, they were taken very seriously by my partner and kind of used as another area of control.” ID11*  *“In that first lockdown, what was different for me because of what I was going through, was you were only allowed out once a day, and I wasn’t allowed out without my son, and it wasn’t really appropriate to take him to the supermarket. So, I had some difficulties because of the abusive situation that was going on…because of the control going on I couldn’t get out for a walk on my own, I couldn’t go to the shops on my own… So, [my partner] would then go to the shops, so I didn’t have control over what I could buy, it was a bit odd.” ID5*  *“He put my health at risk as a clinically extremely vulnerable person. I still think it’s a complete miracle I didn’t catch it. We were doing lots of mitigation to try and prevent that, which shows that the multi-layered approach does work, but he put me at risk.” ID6*  *“The only time it’s been an issue has been if he’s been thinking I haven’t been, in his view, been careful enough… if I walk along the street, I can’t wear a mask, he think[s] I should have worn a mask in the street as well. But I tried to explain I couldn’t see, because of the glasses, but he didn’t really, fully, take that on board, and that was a bit of a bone of contention.” ID4* |
|  | 1. The importance of specific government guidelines for abuse victims and survivors during pandemics | *“It was good hearing things on the news about you don’t have to follow these rules if you’re going through this, it came in quite late, perhaps too late… I kept thinking it’s all very well people saying you have to stay in your home, and stay with people you live with. I’m living with somebody who’s so vile to me, why am I not allowed to see anybody else, safely. I guess we know that now, that can be done outside. And we didn’t know, I don’t blame anyone for that, but that was very, very hard.” ID5*  *“This is really probably to the government for making the decisions on setting the rules, that to be trapped where abuse happens and not have the freedom to go somewhere, other places, is really difficult.” ID2*  *“I see a lot more of my family because I’ve moved area. I think, in the second lockdown, I was able to bubble with my parents because I’m a single parent, so I saw a lot more of them then as well, which was nice.” ID12* |
| Increased feelings of fear, isolation, and loss of control | 1. Feelings of loneliness and isolation that collided with the pandemic and abuse | *“I was in the house for the best part of eight months, last year… until I think, again, maybe August. I think there was the end of a lockdown, wasn’t there, in July last year… it was like we were let out from prison. So, yes, there was a flurry of activity in August and September, and then as it got close to Christmas, I think we all realised that something’s going to change again… So, for me, the main thing, really, is just not being able to be around people that I would have been around, that is the main issue.” ID8*  *“I think I did have this empty space, when my ex left. Empty, like I’d spent a lot of time worrying about this relationship, for the past five years, and expending a lot of energy on it…, a fourth of my brain was probably engaged in dealing with that situation, and now that situation was gone. And then, [Location] locked down, I’m here, I have this empty space, what do I do with it? And I didn’t know how to deal with it… So, that was my sort of thing, and then this whole I’d left this abusive relationship, and the post-abuse situation… But mine exactly coincided with the pandemic.” ID19* |
|  | 1. The pandemic took away certainty and control | *“I think one of the most difficult things when you have experienced domestic abuse is your life is out of your control. So, somebody else is trying to take control. And the more you try and take control back, it actually becomes more frightening because the abuser gets worse… So, with the lockdown, a pandemic situation, I was here. I have no idea where he was. Everybody has to be at home. That doesn't mean to say he was. He’s a criminal, for heaven's sake... and it's that unknown. The unknown is very frightening.” ID2*  *“A lot of people, we met up and then they would ghost me… I had sex in someone’s car, I was doing all of this crazy shit… I was homeless, and a lot of people were being like, you’re homeless and you’re doing shit like this. I don’t know, in a way, I felt like I couldn’t control it.” ID19*  *“I was in such a vulnerable place, without realising it, that I was just pleased to have that kind of opportunity to have a relationship with somebody that wasn’t two feet away from them. Literally, when I look at it now, it really makes sense, because I think if it had been last year, I wouldn’t have done what I did. I think that that’s what it was, I felt vulnerable and alone, and as people say, sometimes negative attention’s better than none.” ID8* |
|  | 1. Risks from domestic abuse far worse than fear of being exposed to covid | *“...I think because of what I’d been through with my ex-partner being so horrible, I was like, oh, what’s a pandemic in relation to that, it really didn’t feel as scary or significant as the other stuff that was going on.”* ID20  *“During the pandemic, you’re naturally more isolated from your friends and family, which is something my partner did anyway, was to isolate me from my friends and family, move me across the country, away from everybody.” ID12*  *“When we went into the first lockdown, I was still strong. People were going, ‘oh God, this is awful.’ I just said, ‘welcome to my world.’ This is what I've lived like… Locked in, not seeing anybody, keeping my distance… I have self-isolated [for years] to be safe, mentally, for my own self.” ID2* |
| Psychosocial wellbeing underwent a transformation during the pandemic | 1. Reconnecting with sense of self after abuse ended | *“Living with him was very draining, I had to do a lot of work because he was incapable of being an adult by himself. So, I’ve been able to focus more on- (pause) Focus is the wrong word, just be myself more with less distractions.” ID20*  *“I had never been in a relationship before my marriage, and the marriage was so unfulfilling, I was also dating, during the pandemic. Which is tricky, for anyone, and it was tricky, for me, because I was in rebound and not just in rebound, but also trying to make up for lost experiences, trying to regain my sexuality, and trying to not lose hope that there are good people out there.”* ID19  *“I’ve made it through that year of being on my own, in my own place, and I’ve made it through the seven months before that, of horrific pressure.” ID7*  *“I think the word that for me characterises my learning from this experience is freedom. And I think freedom can be in lockdown because it's my freedom to do what I want. Even though within the limitation that I'm allowed to, but I'm still free to do what I want as a person.” ID1*  *“I’ve become more confident [during the pandemic]. So, I identified myself as quite a down and negative person, and I put that down to all the experiences I was going through, whereas now I feel like I was a little bit of a caterpillar before. And now I’ve come out. I’m not saying I’m a beautiful butterfly, but I’ve come out feeling a lot more free.” ID10* |
|  | 1. Socialising more now for the first time in a long time | *“Because we couldn't do much during COVID, I tend to actually do too much, so like lots of classes and activities and shows and meeting with friends to kind of overcompensate.” ID1*  *“Coming out of the lockdown coincides with coming out of the relationship. I wasn’t allowed to have any sort of social media when I was with my partner, so actually, in the past couple of weeks, I took the steps to go back on Facebook, [after] eight years away from it, everything’s changed. All my friends had got babies, they’ve got everything, and so that has opened up a different sort of world.” ID10*  *“The positive things were the online shift whereas I could attend some of the social events I wouldn’t otherwise be able to attend with a little child. So, if it’s after their sleep or I could sort of manage with her playing by my side and attending an online event. So, that was a good thing where before if it wasn’t online I couldn’t have access to this.” ID9*  *“A good friend set up a weekly quiz that we did, and it was brilliant. And in fact, I’m a single parent now, but being a parent I hadn’t been able to go to the pub and do that for a while, so to be able to do a kind of pub quiz online” ID5*  *“My social life was zero before. Prior to the crime or crimes occurring, I had a very active social life… So, because all that had already stopped…the pandemic was no different. My social life of having a friend round or anything like that was very rare, because I was still recovering… I have started and gradually, I met for the first time last week with two friends. We’re in a WhatsApp group, which I set up. So, I set the things up and it’s almost like you need some control of your own life back. So, I set things up that I’m happy with.” ID2*  *“I try to have better connections with people already, I don’t know if it’s made it easier to do that in the pandemic because other people are doing that as well. It is something being freeing; we just sort of… I don’t know, I feel like it’s easier to talk to strangers now because everyone again has been in the same boat, we all have something in common.” ID20*  *“I think it’s become very compartmentalised. It probably was before, but I was maybe not aware of it as much, maybe it’s become a bit more acute. So, talking to other people about being a single parent, other parents get it, even if they’re not single parents they just get it, that’s hard. Some people who don’t have kids can appreciate from afar that it’s hard, but they don’t get it, because they’re not in that experience. So, I talk to my friend from DV support about DV, and my counsellor. But then other people, I either don’t want to divulge that, or other people don’t get it… “ID13* |
| Pre-existing and pandemic-related barriers to accessing support | 1. Missed opportunities to intervene and unhelpful referrals | *“A lot of [services are] so directed to younger people, I wouldn’t feel comfortable. I looked into what’s available in the past, and just thought, I can support myself better.” ID8*  *“They sent me somewhere else, and they sent me to a couple of other places, one I’m not in critical need enough for, and the other I’m in too critical need for.”* ID20  *“The last time I saw a doctor there, she was really nice… she said, ‘if you ever just want to come in and have a chat, just come in and have a chat.’ And if that had been possible [during lockdown], that would have been my nearest version of having a family member, it was just that one-to-one, it wasn’t therapy, it would have just been nice to go in and say, oh, I’m having a hard time with this. And I would have done that, and there wasn’t that option, and I could have maybe dealt with it a bit sooner.” ID8*  *“With the GP, they don’t like you to go to the doctor. It’s all ‘send a photograph’ and like we’re talking right now. Again, you can’t see my hands. And a lot of a diagnosis of anxiety, you can tell by the hands. So, to me, the GPs don't necessarily get a true picture. “ ID2*  *“In terms of support for me, I felt like a lot of people didn’t pick up on what I was going through because I didn’t have that face-to-face contact with people. I can remember talking to a health visitor about my partner shouting at my baby, and she was like, oh, yes, men can suffer with post-natal mental health as well.” ID12*  *“We can talk all beautiful things about how we think the relationship should be and it all looks nice, but then the actual behaviour which is abusive was not addressed at all. Nobody hears that. What is the point of the social service meeting at all if that is not addressing the behaviour which would happen?” ID9*  *“I think, because it was lockdown, I don’t think there was (pause) Almost even professionals, in their heads, were making more excuses and being like, oh, well, what was normal isn’t normal anymore, so… You know? I think there’s a number of people who I probably hinted at stuff to who didn’t do anything, or missed what I was saying, or didn’t think about it in the right way. Yes.”* ID12 |
|  | 1. Some support options disappeared suddenly during the pandemic | *“Contacting anybody they would say ‘oh, we are overwhelmed, we can’t provide too much support’.” ID9*  *“It was really difficult. I used to go to… domestic violence groups, and meet other women... And we used to have breakfast clubs in the morning… And I loved it. It was the place where I could actually meet other women like me... And then it all went overnight.”* ID14  *“I think it’s because all the team are mums, so I can completely empathise because of the whole home schooling, and all that kind of stuff, everybody’s trying to juggle work with children, children being at home, and home schooling. Like, some letters were missed, or I didn’t get copied into a letter, and then that was quite a risky moment because I didn’t know what had been said. They just kind of dropped the ball a little bit, they dropped some of the plates that were spinning, a couple of them. Yes, so I really felt the impact of that, and then at the same time, it was like, for them they’re potentially dealing with some really vulnerable women, it’s like a life-or-death situation…” ID13*  *“In the second or third lockdown, I didn’t engage with them anymore, because I knew there was nothing they could do. So, what was the point? Everywhere was shut. I still felt like a sitting duck.” ID14* |
|  | 1. Friends and family unable to help because they were following COVID rules | *“Our older neighbours were left really fearful, and suddenly, all of their support went, so I was running round after them as well, make sure they were okay. Yes, it was pretty bad, really, pretty scary times.” ID7*  *“I could not tell my mum, or broken the rules, and she was shielding, it was impossible. Impossible. It wasn’t anybody’s fault, it’s the way it was.” ID5*  *“I asked [my ex-husband] to find somewhere to go. And he phoned round all his friends, and nobody would take him because of the restrictions, we’ve got friends who were really sticking to everything, as we were, everyone had come so far, nobody wanted to breach it, so there was nowhere he could go.” ID5*  *“But yes, I’m either more acutely aware of it, possibly because of the situation with DV, and very much like, I can’t talk to lots of people about this, this is my support, it’s very specific to support. Or maybe it is because what we’re going through is just so immense, people can’t deal with anyone else’s problems that they can’t directly relate to” ID13*  *“All my family were having their own struggles, all my friends were mutual friends with my husband, I couldn’t turn to any of them, and my one friend in [Location], was locked down with her [family], so she couldn’t even speak privately. So, I just went from one day to the next, really, and tried to eat healthy, and tried not to drink too much, that’s all I could do. It’s just been a waiting it out.” ID7*  *“We had neighbours both sides… They must have heard all sorts, and yet they never did anything… We were joining onto their houses, so they must have heard stuff, but yet they did nothing…. And I do wonder, if we weren’t in a lockdown, whether they would’ve thought that behaviour less acceptable and actually thought this isn’t normal. But I think, probably because we were in lockdown, I think a lot of people justified, ‘oh, people are going to be more shouting at each other. Oh, I’m going to hear more noise.”* ID5  *“The neighbours… they’re still very funny about speaking with me. It’s very strange… they wouldn’t be rude to me in public, but they will walk past me in the street…there is that extra level of aloneness, really, that people don’t want to know, they don’t want to help…If they’d seen a man being abusive or hearing it, they’d have probably, most people might have thought about [Intervening] … When I was reading about all these or was going online and hearing about all these idyllic streets of people, around the country, looking after their neighbours. And not one neighbour on my street asked if I was okay… And I thought, at the time, this, again, it shows people like me where we don’t stand in the community, because you’d think, during a pandemic, there would be something.” ID8* |
| Some forms of support were more readily available and accessible during the pandemic | 1. Increased access to formal support during the pandemic | *“The domestic abuse support from the domestic abuse police officer, DAO, used to be face to face, and it is telephone now only, which is definitely better.” ID2*  *“Yes, it was the [redacted] support service. They were the ones that got me into the hostel to begin with. And they were the ones who really pushed the police to move me. And she was the one that would ring me up every day and work out housing… options for me and stuff like that. I couldn’t have done it without her” ID14* |
|  | 1. Support needs being met through comprehensive and innovative interventions | *“They referred me into the organisation that covers my area, and they did a risk assessment based on the abuse. And they were like, oh, you’re still quite high, you’ll get a support worker, so I got a support worker. And they were like, okay, let’s get you a solicitor…they [also] run a weekly programme for women who’ve been through abuse, which basically looks at identifying different types of abuse, I guess, and also then looks at what’s healthy. So, that’s been quite helpful, but then they also just ring for a check-in.” ID12*  *“And this person, God bless her, she was like, no, this is a really dangerous situation, and she completely understood what I was going through, and she was like, no, you’re not going through, forget about your thing, so basically make sure that I got housing. So, I got housing by, I think, 4 PM…. the next morning…Sometimes you just need actual, substantive support, [like] housing. Someone to do the research for you, maybe, if you’re feeling too overwhelmed, or a GP that understands, and is like, okay, maybe you have this problem, and maybe this is a solution, and is not dismissive.”* ID19  *“On social media, a couple of friends who didn’t know what was going on would signpost people to places, websites and stuff, and that felt very supportive. So I think social media could play a part in adverts, I suppose, saying if you need help, here it is, because you can just see that without having to go searching for it.” ID5*  *“So, my breastfeeding WhatsApp group probably… They’re the most supportive ladies, and I think they were very supportive of… I’d say things and be like, oh, this has happened, and I don’t know if it’s normal or not. And they’d be like, no, it’s not the normal, come round. Get out of your house, come round to mine. And one of them was like, I have a spare room, just come and stay with me, you don’t need to live there with him, but I didn’t really feel I could do that. But I felt very supported by them.” ID12*  *“I actually just went onto a Facebook group that I was already in, it was like a mums’ group…it’s much easier to go on a national kind of group, where you can see if there are any of your friends who are part of it, they weren’t. So, I went and asked there, like what do I do, this has happened, this has happened, this has happened, and people were just brilliant.” ID5*  *“When I was in [the supermarket], when I had this incident, those staff were exceptional. They said, we've got a safe room. I just had to say, can you look after my trolley? I’ve just got to leave. They said, are you all right? No, I have a stalker, domestic abuser, and he’s here. They said, don’t worry, we’ll take this, come into our room. And it was their staff room, but they class it as a safe room.” ID2* |
|  | 1. Engaged more with self-help and self-care during the lockdown | *“Before, I didn’t look after myself. Well, I did, but I didn’t prioritise my own self-care. So, prioritising the children, working, obviously in the abusive relationship I was made to prioritise him, which thankfully is not an issue anymore. But the pandemic has made me view the self-care thing better.” ID13*  *“Expecting people to change is silly in and of itself. But expecting that you can change and how you feel about that person. That is the fundamental complete change that is probably damage that cannot be undone. And I didn’t think it could be undone. I wrote down these things that I was going to tell myself every day. Time heals all wounds.... It does not.” ID3*  *“It’s doing mindful things, where your brain is not distracted by the unwanted thoughts… Appreciating sunshine and the weather. I keep a positive diary... So, every day, I ended up putting positive things in it... So, that was good. And again, these are things which I suppose I've carried through from before.”* ID2 |

Note. Some details redacted for privacy using “[ ]” to denote removed information.
